# Joint prosthetic infections: a success story or a continuous concern?

**DOI:** 10.3109/17453670903487016

**Published:** 2009-12-04

**Authors:** Geert H I M Walenkamp

**Affiliations:** Professor of Orthopaedic Surgery, Caphri Research Institute, Maastricht University Medical Centre, the Netherlands

## Introduction

In this issue of Acta Orthopaedica, there are 2 papers dealing with postoperative infections after joint arthroplasty. Stefansdóttir et al. (2009) discuss the timing of the preoperative prophylactic antibiotics and Dale et al. (2009) report a possible increase in the infection rate for total hip arthroplasty in Norway. These papers give us reason to reflect on the question of whether our efforts to prevent surgical site infections are sufficiently effective, and what percentage of infection we should try to achieve as a result of all our preventive measures.

A deep postoperative infection in orthopedic surgery involves bone and biomaterials, and is difficult to heal without removal of the biomaterials. Although the infection rate of 1–2% in clean orthopedic operations is low compared to other kinds of surgery, there is a constant need to maintain the best possible infection prevention. Now and then, there is an episode of outbreak of surgical site infections (SSIs), sometimes with infection rates of more than 4–5%. The causes of such disastrous periods mostly remain unclear, but often the result is that the preventive measures are tightened by the orthopedic surgeons, which often causes irritation and resistance from other workers in the hospital.

The necessarily authoritarian way of protocol control in a hospital is often violated. We have the same experience as Stefansdóttir et al. that hygiene standards seem to worsen. People have a tendency to do their work in the easiest and most convenient way, which may cause a regrettable relaxation of hygiene standards, as also mentioned by Hughes and Anderson (1999).

Many preventive measures to reduce postoperative infections have been investigated. They are based on improvement of the resistance of the host to infection on the one hand (e.g. body temperature, glucose level, antibiotics, nutritional state), and reduction of peroperative contamination of the wound on the other (e.g. disinfection, clean clothing, ultraclean air). The low infection rate in arthroplasties nowadays makes it almost impossible to perform further randomized trials on infection prevention. In the famous study by Lidwell et al. (1987), which investigated the usefulness of clean air as a prevention measure, more than 8,000 joint prostheses were needed. The Dutch randomized trial by Wymenga (1991) compared the deep infection rate between 1 dose versus 1 day (3 doses) of systemic cefuroxim prophylaxis in 2,651 total hip implantations. Even this number was not enough to achieve a statistically significant result (0.83% vs. 0.45%), although the trend was that the 1-dose regimen doubled the infection rate. With such low infection rates, prophylactic studies become so large that they can no longer be financed.

The lack of a high level of evidence from a randomized trial is not, however, proof of ineffectiveness: the absence of evidence is not the evidence of absence. In national guidelines, the level of evidence should be given as has been done, for example, in the CDC guidelines (Mangram et al. 1999). Evidence from experiments and also theories based on the understanding of the “route of infection” should also be taken into account.

In the Netherlands, a quality improvement program run by the CBO (the Dutch Institute for Healthcare Improvement, Utrecht, the Netherlands) has been in existence for 15 years to reduce postoperative infections (CBO 2009). The method of the “plan-do-study-act” (PDSA) cycle was used to improve process parameters without measuring the SSI rates. Accepted preventive measures were subjected to such PDSA approaches, such as limited preoperative shaving with clippers of only the incision site, minimizing the number of door openings during operations (van Tiel et al. 2006), and also the infusion of the prophylactic antibiotic at the right time, as now discussed in the Swedish study by Stefansdóttir. The acceptance of these hygiene improvements in daily OP practice is slow and takes years, but there is a clear tendency. Whether or not this does indeed result in a lower surgical site infection rate is not yet known, and it has now been seriously called into question by the Norwegian register data.

Systemic antibiotics are the best documented—and also the most effective—prophylactic measure to reduce surgical site infections. The reduction rate is about 80% (AlBuhairan et al. 2008). There is no doubt that the timing is crucial: antibiotics must been given intravenously 15–45 min before incision (Manniën et al. 2006). The choice of antibiotic (narrow or broader spectrum) and the dose (1 dose vs. 1 day) is more controversial. In general, the 1-day regimen is better in arthroplasty (Wymenga 1991, Engesaeter et al. 2006), and 1 dose is only effective if the half life of the antibiotic is more then 12 hours (Gillespie and Walenkamp 2001). The disappointing result in the paper from Sweden (Stefansdóttir et al. 2009) that in almost 50% of the operations the timing was not correct, illustrates that there is an urgent need for an involved surgeon at each department who repeatedly checks whether the whole package of preventive measures is being applied and who motivates his or her colleagues to adhere to treatment protocols.

When working on infection prophylaxis, one must know what SSI rate has to be achieved. The infection rate is one of the most important of the many quality parameters that are used for operations. Increasingly, hospital managers are using these data to judge whether departments are underperforming and the data from the national arthroplasty registers can be used in the same way (Robertsson 2007).

In the Netherlands, the government Inspectorate of Healthcare has made it obligatory for surgical departments to organize a reliable infection registration of their operations, and this information is made publicly available. Today, however, insurance companies also ask about data from the infection and complication registrations, and they use these data in their decision on which orthopedics departments and hospitals are contracted to implant prostheses. However, isolated SSI data not related to patient mix may cause misjudgements and incorrect decisions.

The question remains as to what SSI rate is acceptable, and where we can find the best benchmark data. Several national surveillance programs for nosocomial infections exist, which gather data on incidence of SSI. In the Netherlands, the national PREZIES surveillance program has been recording postoperative infections from all types of surgery since 1996. The database of 1999–2008 covers 203,359 operations with 5,985 deep and superficial postoperative infections (2.9%). There are 52,133 total hip operations included, of which only 29,876 were adequately followed with a surveillance after discharge as advised (up to 1 year). The incidence of infection in these patients was 1.0% deep and 1.1% superficial (PREZIES National Surveillance Network for Hospital Infections 2009). In other countries, comparative incidence surveillance programs exist: Germany (NRZ-KISS), Belgium (NSIH), England (NINSS), Austria (ANISS), France, the US (Woodhead et al. 2002), and Australia (VICNISS). Comparisons of results between countries have been published: between the Netherlands and Germany (Manniën et al. 2007), and between England and the USA (Leong et al. 2006). In many countries, about 50% of the data collected apply to orthopedic operations, reflecting the relatively high degree of interest of orthopedic surgeons in infection surveillance.

Because superficial infections are difficult to distinguish from aseptic wound complications and are often treated by family doctors after discharge, their registration is not reliable and it is better to focus on deep infections only. Surveillance after discharge for up to 1 year, as suggested by the the CDC, is important (Mangram et al. 1999). This minimum follow-up time at the outpatient clinic requires both involvement and organizational abilities on the part of orthopedic surgeons (Walenkamp 2003).

In the several national incidence surveillance programs, there is no indication that the deep infection rate for total hip arthroplasty is increasing: for many years it has remained around 1%. The Dutch data show a statistically significant decrease of 60% (Manniën et al. 2008), as mentioned by Stefansdóttir. In this calculation, however, superficial and deep infections were pooled. If only the deep infections are considered in the Dutch PREZIES registration, there appears to have been no statistically significant change in the infection rate in 10 years (van Benthem and Manniën 2009)

The question is whether arthroplasty registers can be used to analyze trends in postoperative infections. As with most other registers, the arthroplasty register in Norway gives information mainly based on the registration of revisions with removal or exchange of the whole or a part of a prosthesis (Engesaeter et al. 2006, Helse-Bergen 2008, Kärrholm et al. 2008, Hooper et al. 2009). If a reoperation is necessary without removal or exchange, then it is not recorded.

Early postoperative prosthesis infections should be treated in the first postoperative weeks. With a combination of aggressive surgical debridement with the prosthesis in situ and high-dose antibiotics, most infected prostheses can be saved, in total hips nowadays up to 70% (Crockarell et al. 1998, Guilieri et al. 2004, Trebse et al. 2005, Toms et al. 2006). These infected but retained prostheses, treated in situ without removal, are not recorded as infected in a register that is based on removal or exchange of prosthesis parts. Thus, an additional registration of such an early reoperation is necessary—as recently introduced in the Swedish and Finnish registers, for example.

In the Swedish register, the reoperations are subdivided into 3 groups: (1) revision with replacement or extraction of implant components, (2) major reoperations without replacement or removal, and (3) minor reoperations without replacement or removal. In this register, the number of reoperations in 2006 and 2007 increased by 2.7%, and for deep infections the number increased by 6.6% (Kärrholm et al. 2008). The percentage of reoperations for infections within the first 2 postoperative years nationwide in Sweden was 0.6%, with a range between the hospitals of 0.0–2.8%. These data do not, however, capture postoperative infections that were not treated surgically. If these would have been included, the total infection rate for total hips in Sweden would appear to approach the 1% level, as found in the surveillance programs mentioned.

It has been stated that reoperation within 2 years “reflects mainly early and serious complications such as deep infections and revision due to repeated dislocation” and “is a quicker quality indicator and is easier to use in clinical improvement work than 10-year survival, which is an important but slow and historical indicator” (Kärrholm et al. 2008).

The Finnish Knee Arthroplasty Register (FAR) met the same problem in their study of knee prosthesis infections in the past few years. Jämsen et al. (2009) reviewed 38,676 knee prosthesis operations but they used not only revisions but also reoperations as endpoint. Because they supposed that many infection-related operations such as debridement, amputation, and arthrodesis were infrequently reported to the FAR, they collected parallel information from the Finnish Hospital Discharge Register (HDR), which gives better information based on diagnoses. Comparison of the 2 databases gave information about the reoperated infected prostheses, but not about the infections that were only treated with systemic antibiotics. These authors confirmed that the Finnish register underestimates the infection rate.

In conclusion, there are 3 levels of registration available in large databases with an increasing degree of reliability: firstly, registrations of revisions for infection with component removal or exchange, then reoperations for infection but with retention of the prosthesis, and finally surveillance programs on incidence of surgical site infection in departments, hospitals, or countries. These combined data should be used for reliable estimation of the true infection rate.

The report from the Norwegian register of a probable increase in the percentage of total hip prostheses that had to be removed because of infection is interesting, but the reason for the increase is unclear. The authors' analysis is relevant, but I would like to add the possibility that the increase in more resistant germs such as MRSE and MRSA, and the technically more complex reconstructions have resulted in infections that are more difficult to treat. So the question remains whether the infection rate in total hips increases.

In 2001, Lidgren, co-author of the paper by Stefansdótter et al., wrote a guest editorial in this journal on the same subject with the optimistic title: “Joint prosthetic infections: a success story” (Lidgren 2001). They now suggest in their own article that this statement is no longer true, and that the problem remains as before.

There is an indication that prophylactic hygiene standards in hospitals should be improved. There is also a need for more exact data on infection rates, perhaps by a smart combination of data provided by the increasing number of arthroplasty registers and by national SSI surveillance programs. We must not be satisfied with a deep infection rate of more then 1% for clean orthopedic operations, and we must be able to prove that relatively low infection rate using reliable surveillance.

## References

[CIT0001] AlBuhairan B, Hind D, Hutchinson A (2008). Antibiotic prophylaxis for wound infections in total joint arthroplasty. A systematic review. J Bone Joint Surg (Br).

[CIT0002] CBO http://www.cbo.nl/english/default_view 2009.

[CIT0003] Crockarell JR, Hanssen A D, Osmon DR, Morrey B F (1998). Treatment of infection with debridement and retention of the components following hip arthroplasty. J Bone Joint Surg (Am).

[CIT0004] Dale H, Hallan G, Espehaug B, Havelin L, Engesaeter L B (2009). Increasing risk for revision due to deep infection after total hip arthroplasty. A study on 97,344 primary total hip replacements in the Norwegian Arthroplasty Register from 1987 to 2007. Acta Orthop.

[CIT0005] Engesaeter LB, Espehaug B, Lie SA, Furnes O, Havelin L (2006). Does cement increase the risk of infection in primary total hip arthroplasty? Revision rates in 56,275 cemented and uncemented primary THAs followed for 0-16 years in the Norwegian Arthroplasty Register. Acta Orthop.

[CIT0006] Gillespie WJ, Walenkamp G (2001). Antibiotic prophylaxis for surgery for proximal femoral and other closed long bone fractures. Cochrane Database Syst Rev.

[CIT0007] Guilieri SG, Graber P, Ochsner PE, Zimmerli W (2004). Management of infection associated with total hip arthroplasty according to a treatment algorithm. Infection.

[CIT0008] Helse-Bergen HF (2008). The Norwegian Arthroplasty Register. http://www.haukeland.no.nrl.

[CIT0009] Hooper GJ, Rothwell AG, Stringer M, Frampton C (2009). Revision following cemented and uncemented primary total hip replacement. J Bone Joint Surg (Br).

[CIT0010] Hughes S, Anderson F (1999). Infection in the operating room. Editorial. J Bone Joint Surg (Br).

[CIT0011] Jämsen E, Huotari K, Huhtala H, Nevalainen J, Konntinen YT (2009). Low rate of infected knee replacements in a nationwide series—is it an underestimate? Review of the Finnish Arthroplasty Register on 38,676 operations performed in 1997 through 2003. Acta Orthop.

[CIT0012] Kärrholm J, Garellick G, Rogmark C, Herberts P (2008). Swedish Hip Arthroplasty Register. Annual Report 2007.

[CIT0013] Leong G, Wilson J, Charlett A (2006). Duration of operation as a risk factor for surgical site infection: comparison of English and US data. J Hosp Infect.

[CIT0014] Lidgren L (2001). Joint prosthetic infections: A success story. Acta Orthop Scand.

[CIT0015] Lidwell O, Elson R, Lowbury E, Whyte W, Blowers R, Stanley S, Lowe D (1987). Ultraclean air and antibiotics for prevention of postoperative infection. A multicenter study of 8,052 joint replacement operations. Acta Orthop Scand.

[CIT0016] Mangram AJ, Horan TC, Pearson ML, Silver LC, Jarvis WR, van Belkum A (1999). Guideline for prevention of surgical site infection, 1999. Infect Control Hosp Epidemiol.

[CIT0017] Manniën J, Kasteren van M, Nagelkerke N, Gyssens I, Kullberg B-J, Wille JC, de Boer A (2006). Effect of optimized antibiotic prophylaxis on the incidence of surgical site infections. Infect Control Hosp Epidemiol.

[CIT0018] Manniën J, van den Hof S, Brandt C, Behnke M, Wille JC, Gastmeier P (2007). Comparison of the National Surgical Site Infection surveillance data between The Netherlands and Germany: PREZIES versus KISS. J Hosp Infect.

[CIT0019] Manniën J, van den Hof S, Muilwijk J, van den Broek P, Benthem B, Wille JC (2008). Trends in the incidence of surgical site infection in the Netherlands. Infect Control Hosp Epidemiol.

[CIT0020] PREZIES National Surveillance Network for Hospital Infections (2009). http://www.prezies.nl/zkh/powi/ref_cijfers.html.

[CIT0021] Robertsson O (2007). Knee arthroplasty registers. Review article. J Bone Joint Surg (Br).

[CIT0022] Stefansdóttir A, Robertsson O, W-Dahl A, Gustafson P, Lidgren L (2009). Inadequate timing of prophylactic antibiotics in orthopedic surgery. We can do better. Acta Orthop.

[CIT0023] Toms AD, Davidson D, Masri BA, Duncan CP (2006). The management of peri-prosthetic infection in total joint arthroplasty. J Bone Joint Surg (Br).

[CIT0024] Trebse R, Pisot V, Trampuz A (2005). Treatment of infected retained implants. J Bone Joint Surg (Br).

[CIT0025] Walenkamp GHIM (2003). Surveillance of surgical-site infections in orthopedics. Guest editorial. Acta Orthop.

[CIT0026] van Benthem B, Manniën J (2009).

[CIT0027] van Tiel FH, Elenbaas T WO, Voskuilen B, Herczeg J, Verheggen FW, Mochtar B, Stobberingh EE (2006). Plan-do-study-act cycles as an instrument for improvement of compliance with infection control measures in care of patients after cardiothoracic surgery. J Hosp Infect.

[CIT0028] Woodhead K, Taylor EW, Bannister G, Chesworth T, Hoffman P, Humphreys H (2002). Behaviours and rituals in the operating theatre. A report from the Hospital Infection Society Working Party on infection control in operating theatres. J Hosp Infect.

[CIT0029] Wymenga A (1991). Joint sepsis after prophylaxis with one or three doses of cefuroxime in hip and knee replacement surgery.

